# Functional Analysis of an S-Layer-Associated Fibronectin-Binding Protein in Lactobacillus acidophilus NCFM

**DOI:** 10.1128/AEM.00024-16

**Published:** 2016-04-18

**Authors:** Jeffrey P. Hymes, Brant R. Johnson, Rodolphe Barrangou, Todd R. Klaenhammer

**Affiliations:** a Department of Food, Bioprocessing, and Nutrition Sciences, North Carolina State University, Raleigh, North Carolina, USA; b Graduate Program in Microbiology, College of Agriculture and Life Sciences, North Carolina State University, Raleigh, North Carolina, USA; Pennsylvania State University

## Abstract

Bacterial surface layers (S-layers) are crystalline arrays of self-assembling proteinaceous subunits called S-layer proteins (Slps) that comprise the outermost layer of the cell envelope. Many additional proteins that are associated with or embedded within the S-layer have been identified in Lactobacillus acidophilus NCFM, an S-layer-forming bacterium that is widely used in fermented dairy products and probiotic supplements. One putative S-layer-associated protein (SLAP), LBA0191, was predicted to mediate adhesion to fibronectin based on the *in silico* detection of a fibronectin-binding domain. Fibronectin is a major component of the extracellular matrix (ECM) of intestinal epithelial cells. Adhesion to intestinal epithelial cells is considered an important trait for probiotic microorganisms during transit and potential association with the intestinal mucosa. To investigate the functional role of LBA0191 (designated FbpB) in L. acidophilus NCFM, an *fbpB*-deficient strain was constructed. The L. acidophilus mutant with a deletion of *fbpB* lost the ability to adhere to mucin and fibronectin *in vitro*. Homologues of *fbpB* were identified in five additional putative S-layer-forming species, but no homologues were detected in species outside the L. acidophilus homology group.

## INTRODUCTION

Fibronectin is a glycoprotein, which functions as an essential link between cells and extracellular matrices (1). Known to play an important role in the regulation of cell adhesion, migration, and tissue repair, fibronectin molecules are produced by a variety of cell types, including intestinal epithelial cells (2). After secretion by the cell, fibronectin molecules bind to transmembrane integrins in the extracellular matrix (ECM) (3). Integrin binding facilitates fibronectin dimerization, allowing fibronectin to bind other extracellular proteins. Fibronectin-binding proteins (FnBPs), which bind specifically to fibronectin, are found across a wide variety of Gram-positive and Gram-negative bacteria, both pathogens and commensals (1, 3). Bacterial species often have multiple diverse FnBPs. Although distinct subgroups of FnBPs have been identified, no common features have been detected among the vast array of known FnBPs. The most prevalent subgroups of FnBPs have been characterized (1, 4–8).

Lactobacilli are Gram-positive rod-shaped lactic acid bacteria. Many Lactobacillus species are indigenous to fermented foods and food-related habitats, including the mucosal surfaces of animals. Comparative genomic analyses of similar species from plant, dairy, and animal habitats illustrate the ability of members of the genus Lactobacillus to adapt to diverse environments (9). Lactobacilli are normal components of the gut microbiome, with many strains considered to be probiotics (10). Probiotic bacteria have been shown to confer positive health benefits to the host through immunomodulation (11–16) and pathogen exclusion (17–20). Thus, adherence to intestinal epithelial cells, mediated by extracellular proteins, is widely regarded as one important feature of probiotic efficacy (21–23). Select species of Lactobacillus are known to form a unique self-assembling crystalline array of protein on the outermost layer of the cell (24, 25). This array, known as a surface layer (S-layer), is an important factor in cell morphology and adhesion to intestinal epithelial cells (26).

The S-layer-forming bacterium Lactobacillus acidophilus NCFM has been studied extensively as a probiotic (27, 28). The major component of the S-layer in L. acidophilus is the 46-kDa protein SlpA (29). Inactivation of SlpA has been shown to drastically reduce adhesion to Caco-2 intestinal epithelial cells (30). A protein with significant homology to Fbp54 of Streptococcus pyogenes has been identified in L. acidophilus NCFM (1, 31). The deletion of FbpA from L. acidophilus NCFM has been shown to reduce adhesion to Caco-2 epithelial cells by 76% (30). FbpA homologues are widespread in Lactobacillus genomes (1, 32).

A second fibronectin-binding protein in L. acidophilus NCFM, LBA0191, was bioinformatically predicted based on the presence of a fibronectin type III domain (FN3). FN3 domains are commonly found in a diverse subset of animal and bacterial proteins, including cell surface receptors, muscle proteins, and extracellular matrix molecules (33–35). The protein also contains a putative signal peptide cleavage site at the N terminus that marks LBA0191 for secretion from the cell, as well as a central collagen-binding domain (36). LBA0191 was recently identified as an S-layer-associated protein (SLAP) (37). SLAPs are proteins that may be associated with or embedded within the S-layer complex (37). Taken together, these observations suggest that LBA0191 may be involved in the binding of fibronectin.

To determine the function of LBA0191, the *lba0191* gene was deleted from the chromosome of L. acidophilus NCFM using a *upp*-based counterselective gene replacement system (38). In the present study, we phenotypically characterized this strain (NCK2393) in order to investigate the role of LBA0191 in adhesion to fibronectin, mucin, and collagen. We provide evidence that LBA0191, designated FbpB, plays a significant role in adhesion to fibronectin and mucin *in vitro*. Bioinformatic evidence further indicates that the function of FbpB may be conserved among homologues in closely related lactobacilli.

## MATERIALS AND METHODS

### Bacterial strains and growth conditions.

The bacterial strains, plasmids, and primers used in this study are listed in Table 1. Strains of L. acidophilus were propagated statically in de Man-Rogosa-Sharpe (MRS) broth (Difco Laboratories, Inc., Detroit, MI) or on MRS agar (1.5% [wt/vol]; Difco) under aerobic conditions at 37°C or 42°C. Transformants were selected in the presence of 2 μg/ml erythromycin (Em) (Sigma-Aldrich, St. Louis, MO) and/or 2 to 5 μg/ml chloramphenicol (Cm) (Sigma). Escherichia coli was grown in brain heart infusion (BHI) medium (Difco) at 37°C with shaking aeration. E. coli EC101 was propagated in the presence of 40 μg/ml kanamycin (Km) (39). For *upp*-based counterselective gene replacement procedures, plasmid-free double recombinants were selected on a glucose semidefined agar medium containing 100 μg/ml 5-fluorouracil (5-FU) (Sigma), as previously described (38).

**TABLE 1:** Strains, plasmids, and primers used in this study

Strain, plasmid, or primer	Genotype, characteristics, or sequence^*a*^	Source or reference
Strains		
*L. acidophilus*		
NCFM	Human intestinal isolate	Sanders & Klaenhammer (27)
NCK1909	NCFM carrying a 315-bp in-frame deletion within *upp* gene	Goh et al. (38)
NCK1910	NCK1909 harboring pTRK669; host for pORI-based counter selective integration vector	Goh et al. (38)
NCK2393	NCK1909 carrying a 1.2-kb in-frame deletion within the *fbpB* gene	This study
E. coli		
EC101	RepA^+^ JM101; Km^r^ Em^r^; *repA* from pWV01 integrated in chromosome; host for pORI-based plasmids	Law et al. (39)
Plasmids		
pTRK669	Ori (pWV01); Cm^r^; RepA^+^; thermosensitive	Russell & Klaenhammer (56)
pTRK935	3.0 kb; pORI28 with a *upp* expression cassette and *lacZ′*	Goh et al. (38)
pTRK1095	4.1 kb; pTRK935 integration vector with flanking regions of *fbpB* cloned into BamHI/SacI sites	This study
Primers		
0191-1	GTAATAGGATCCAAGCACTTTTGACTGAAGTA	This study
0191-2	TGCCTTTAGCTACATAGTTG	This study
0191-3	CTATGTAGCTAAAGGCAGGTCAACTGTAAAGGAAGTT	This study
0191-4	TAAAGTAGAGCTCCTTCGTTATGCTTAACTTGT	This study
0191-UP	AGCCACTCTGCGTTGTTTCT	This study
0191-DN	TGCAAGAGAACATGGTGCTAA	This study
0191-delcon-F	AATGACAATGGCACTTGCTG	This study

a For primers, the 5′–3′ sequences are given. Restriction enzyme sites are underlined. Km^r^, kanamycin resistance; Em^r^, erythromycin resistance; Cm^r^, chloramphenicol resistance.

### DNA manipulation and transformation.

Genomic DNA from L. acidophilus was isolated using a Zymo Research fungal/bacterial DNA MiniPrep kit. Plasmid DNA from E. coli was isolated using the QIAprep spin miniprep kit (Qiagen). Restriction enzyme digestion was performed using Roche restriction enzymes (Roche Diagnostics). Ligations were performed using T4 DNA ligase (New England BioLabs). PCR primers were designed based on genomic sequence data and synthesized by Integrated DNA Technologies. PCRs were carried out in Bio-Rad MyCycler thermocyclers (Bio-Rad Laboratories) using Choice-*Taq* Blue DNA polymerase (Denville Scientific) for screening of recombinants and *PfuUltra* II fusion HS DNA polymerase (Agilent Technologies) for cloning purposes. PCR amplicons were analyzed on 0.8% agarose gels and purified using QIAquick gel extraction kits (Qiagen). DNA sequencing was performed by Eton Bioscience (Durham, NC).

### Genomic *in silico* analysis.

Genomes were curated from the genome library of the National Center for Biotechnology Information (NCBI) under accession numbers NC_006814.3, NC_021744.1, NC_014724.1, and NC_014106.1. Sequences were compared using the BLASTN and BLASTP features of NCBI (40). Signal peptidase cleavage sites for protein sequences were predicted using SignalP 4.1 (41). Domains were predicted using the NCBI Conserved Domain Database (42). Full genomes and protein sequences were uploaded to Geneious 7.1.9 for comparative genomic analyses (43). Alignments of protein sequences were performed in Geneious 8.1.7 using Clustal W with the BLOSUM cost matrix, and clustering was performed by the neighbor-joining method (44). Protein domains were predicted within Geneious using the InterProScan plugin (45).

### RNA sequence analysis.

RNA sequencing analysis was performed on data from a previous study (46). Transcriptomes of L. acidophilus NCFM, Lactobacillus crispatus ST1, Lactobacillus amylovorus GRL1112, and Lactobacillus helveticus CNRZ32 were analyzed using Geneious version 8.0.5 (43). The expression level calculator within Geneious version 8.0.5 was used to determine normalized transcripts per million (TPM).

### Construction of an L. acidophilus Δ*lba0191* mutant.

A *upp*-based counterselective gene replacement system (38) was used to create an internal deletion of a 1,218-bp region containing *lba0191* (1,403 bp) from NCK1909, a *upp*-deficient background strain of L. acidophilus NCFM. First, 442-bp and 592-bp DNA segments flanking the regions upstream and downstream of the deletion target, respectively, were amplified using two sets of primers. The upstream and downstream regions were amplified with primers (Table 1). The two purified PCR products were fused using splicing by overlap extension PCR (SOE-PCR) and further amplified to construct the Δ*lba0191* allele. Two restriction sites, BamHI and SacI, were engineered onto the upstream and downstream ends of the deletion construct, respectively. The construct was digested with BamHI and SacI and ligated into the multiple-cloning site of a similarly digested integration plasmid, pTRK935. The plasmid containing the deletion construct, pTRK1095, was transformed into competent E. coli EC101. The resulting recombinant plasmid was electroporated into NCK1910, the L. acidophilus Δ*upp* host strain containing helper plasmid pTRK669 (56). Plasmid-free recombinants were recovered, as previously described (38). Double recombinants with the Δ*lba0191* allele were recovered and screened by colony PCR using primers 0191-UP and 0191-DN. Sequence integrity was confirmed by sequencing with primers 0191-delcon-F and 0191-DN (Table 1).

### Stress challenge assays.

Strains were grown to early log phase in MRS broth (∼3 h at 37°C to an optical density at 600 nm [OD_600_] of 0.25 to 0.3) before they were subjected to stress challenges (47). Cells were inoculated into a 96-well plate containing 200 μl per well of (i) MRS broth, (ii) MRS broth with 10% (vol/vol) ethanol, (iii) MRS broth with 0.3% (wt/vol) oxgall bile (Difco), (iv) MRS broth with 2.5% (wt/vol) NaCl, or (v) MRS broth with 0.02% (wt/vol) sodium dodecyl sulfate (SDS). Growth was measured by OD_600_ over a 48-h incubation period at 37°C using a FLUOStar Optima microtiter plate reader (BMG Labtech, Cary, NC). Viable cell counts were determined by diluting and plating onto MRS agar after 1- or 2-h incubation periods at 37°C.

### Exposure to simulated gastric juice.

Simulated gastric juice assays were performed as described previously (48). Overnight cultures were centrifuged (1,771 × *g*, 15 min, room temperature), washed twice, and resuspended in 1.4 ml of sterile distilled water. Simulated gastric juice (0.5% [wt/vol] NaCl solution with 3 g/liter pepsin [pH 2]) was prepared on the day of the experiment. Six milliliters of simulated gastric juice was added to the cell suspension, and viable cell counts were determined by plating in duplicate at 30-min intervals over 90 min. The assay was performed with three independent cultures for each strain.

### ECM adherence assays.

Mucin (type III from porcine stomach; Sigma) was dissolved in phosphate-buffered saline (PBS) to a final concentration of 10 mg/ml. Fibronectin (from human plasma; Sigma) and collagen (type IV from human cell culture; Sigma) were dissolved in 50 mM carbonate-bicarbonate buffer (pH 9.6) (Sigma) to a final concentration of 10 μg/ml. For each assay, a Nunc MaxiSorp 96-well microplate was coated with 100 μl/well substrate and incubated at 4°C overnight. The wells were washed twice with PBS to remove excess substrate before being blocked with 150 μl of 2% bovine serum albumin (BSA) solution (Invitrogen) for 2 h at 37°C. Excess BSA was removed by two additional washes with PBS.

Bacterial cells were grown in MRS to stationary phase in preparation for the adherence assay. Cultures were centrifuged (1,771 × *g*, 15 min, room temperature), washed once, and resuspended in PBS before the cell density was adjusted to ∼1 × 10^8^ CFU/ml, based on the OD_600_. Cell suspensions (100 μl) were added to each protein-coated well. Initial cell counts were enumerated on MRS plates. After incubation for 1 h at 37°C, the wells were gently washed five times with 200 μl/well PBS. Adhered cells were recovered by adding 100 μl of 0.05% Triton X-100 solution (prepared in PBS; FisherBiotech) to each well and agitating on an orbital shaker (200 rpm) for 15 min. Cell suspensions were transferred into 900 μl of 0.1× MRS before being further diluted and plated in duplicate on MRS plates. After growth, colonies were enumerated and expressed as a percentage of relative adherence (mutant CFU/parent CFU), in which the parent CFU quantity was defined as 100%. Adherence assays were performed with at least 3 technical replicates and 3 independent cultures per strain.

### MATS assay.

The assay for microbial adhesion to solvents (MATS) was performed as described by Bellon-Fontaine et al. (49), with four solvents: chloroform (Lewis acid, electron acceptor, polar), hexadecane (nonpolar), ethyl acetate (Lewis base, electron donor, polar), and decane (nonpolar). Cultures were grown in MRS broth at 37°C to stationary phase and harvested by centrifugation (3,220 × *g*, 15 min, room temperature) and washed twice with PBS. Cells were resuspended in PBS to approximately 10^8^ CFU/ml, and the optical density at 400 nm (OD_400_) was measured. Next, 1.2 ml of each cell suspension was mixed with 200 μl of each solvent in a glass round-bottom tube. The mixture was agitated for 60 s and incubated at room temperature for 15 min. After complete separation of the two phases, the aqueous phase was removed, and the OD_400_ was measured. Affinity of the cells to each solvent was calculated with the following equation: 100 × [1 − (*A/A*_0_)], where *A*_0_ and *A* represent the OD_400_ of the cell suspension before and after mixing with the solvents, respectively. Assays were performed in biological triplicate.

### SEM/TEM.

Cells were grown in MRS (35 ml) to logarithmic and stationary phases. The cells were pelleted by centrifugation at 3,166 × *g* for 15 min at room temperature. The cell pellets were resuspended in a fresh 1:1 (vol/vol) fixative mixture of 6% glutaraldehyde and 0.2 M sodium cacodylate (pH 5.5) and stored at 4°C. Sample processing for scanning electron microscopy (SEM) and transmission electron microscopy (TEM) was performed by the Center for Electron Microscopy at North Carolina State University, Raleigh, NC. SEM samples were viewed with a JEOL JSM 5900LV scanning electron microscope at 15 kV. TEM samples were viewed with a JEOL 100S transmission electron microscope. Surface layer thickness was measured with a ruler (in milliliters) scaled to the micrograph scale bar (in micrometers or nanometers) for each image. The sample size ranged from 20 to 29 individual cells for each strain at each phase.

### Extraction of noncovalently bound cell surface proteins.

Noncovalently bound cell surface proteins, including S-layer proteins (Slps) and S-layer-associated proteins (SLAPs), were extracted from L. acidophilus strains (NCK1909 and NCK2393) using LiCl denaturing salt, as described previously (37). Briefly, cells were grown in 200 ml of MRS to stationary phase (16 h), centrifuged (1,771 × *g*, 10 min, 4°C), and washed twice with 25 ml of cold PBS (pH 7.4). The cells were agitated for 15 min at 4°C following the addition of 5 M LiCl (Fisher). Supernatants containing Slps and SLAPs were harvested via centrifugation (8,994 × *g*, 10 min, 4°C), transferred to a 6,000- to 8,000-kDa Spectra/Por molecular porous membrane (Spectrum Laboratories), and dialyzed against cold distilled water for 24 h. The precipitate was harvested at 20,000 × *g* for 30 min and agitated for a second time with 1 M LiCl for 15 min at 4°C to dissociate the SLAPs from the Slps. The suspension was centrifuged at 20,000 × *g* for 10 min, and the SLAP supernatants were separated from the Slp pellet. The remaining suspension was transferred to the 6,000- to 8,000-kDa Spectra/Por molecular porous membrane and dialyzed against cold distilled water for 24 h. Finally, the precipitate was harvested via centrifugation (20,000 × *g*, 30 min, 4°C) to pellet the SLAPs. Both Slp and SLAP pellets were resuspended in 10% (wt/vol) SDS (Fisher). Proteins were quantified via the bicinchoninic acid (BCA) assay kit (Thermo Scientific) and visualized via SDS-PAGE with precast 4 to 20% Precise Tris-HEPES protein gels (Thermo Scientific). Gels were stained using AcquaStain (Bulldog Bio), according to the manufacturer's instructions.

## RESULTS

### *In silico* analysis of *fbpB*.

**FIG 1:**
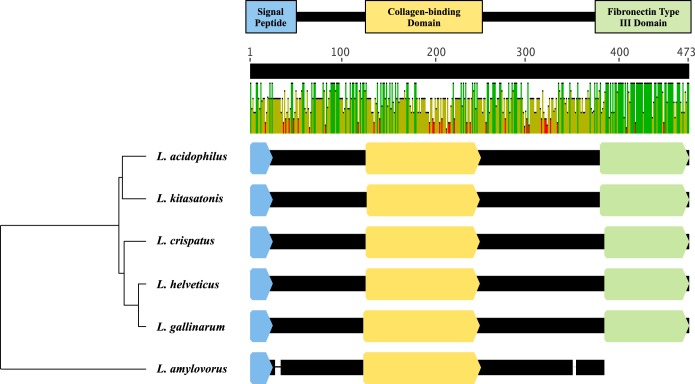
Amino acid sequence alignment of FbpB homologues in Lactobacillus species. The colored boxes represent domains predicted by InterProScan 5. The bar graph shows the mean pairwise identity across homologues at each position in the amino acid alignment (positions 1 to 473, labeled at top). Green bars indicate 100% identity, yellow bars indicate 30 to 100% identity, and red bars indicate <30% identity.

Bioinformatic analysis of L. acidophilus NCFM led to the identification of *fbpB* (*lba0191*), which was predicted to encode a fibronectin-binding protein. This protein was 463 amino acids in length and contained a putative C-terminal fibronectin type III domain (FN3) (cd00063) with a cytokine receptor motif (Fig. 1). A signal peptidase cleavage site was predicted between residues 24 and 25 (VQA/GT) of the hydrophobic N terminus. The 52-kDa protein has a basic isoelectric point (pI, ∼9.6) and a predicted GRAVY value of −0.589. No transmembrane domains were detected in the protein sequence of FbpB from L. acidophilus NCFM.

Analysis with BLASTP shows that *fbpB* from L. acidophilus shares a high level of sequence homology with genes from other species of the Lactobacillus delbrueckii/L. acidophilus complex (Fig. 1) (50, 51), including Lactobacillus kitasatonis (84% identity, GenBank accession no. WP_025014788), Lactobacillus gallinarum (83% identity, GenBank accession no. WP_025005703), L. helveticus (79 to 82% identity, GenBank accession no. CP002081), and L. crispatus (79% identity, GenBank accession no. NC_014106). Additional sequences with low levels of homology to *fbpB* are found in L. amylovorus (35% identity, GenBank accession no. CP002338).

**FIG 2:**
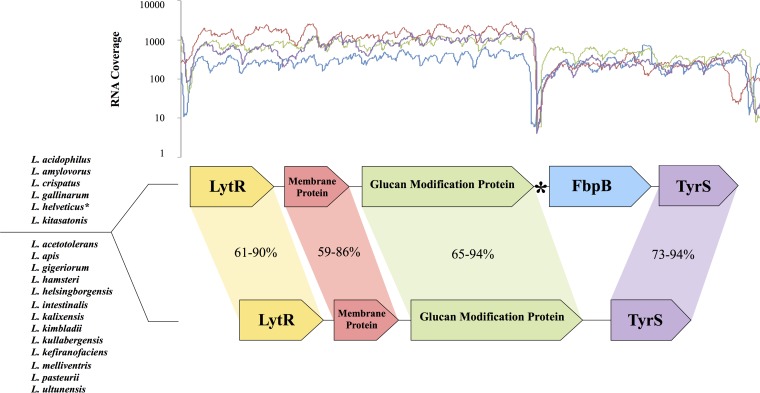
Conserved genomic context of FbpB homologues in the genomes of closely related lactobacilli (top group of species). The transcriptional profile graph shows RNA coverage over the contextual region of four species with FbpB homologues (blue, L. acidophilus NCFM; red, L. amylovorus GRL1112; green, L. crispatus ST1; purple, L. helveticus CNRZ32). The genome of L. helveticus (asterisk) contains a putative 1.3-kb mobile genetic element between the glucan modification protein and FbpB. The region of the transcriptional profile corresponding to the mobile genetic element was deleted from L. helveticus CNRZ32 to allow for comparison to other species. FbpB is absent from all other lactobacilli, although the genomic context is conserved in many species (bottom group of species). The percent identity values indicate amino acid sequence similarity of respective open reading frames (ORFs).

Three genes immediately upstream of *fbpB* and one gene immediately downstream of *fbpB* were conserved among all occurrences of homologues (Fig. 2). In contrast, this genomic context was present with no medial *fbpB* homologue in the genomes of closely related lactobacilli, including L. acetotolerans, L. apis, L. gigeriorum, L. hamsteri, L helsingborgensis, L. intestinalis, L. kalixensis, L. kimbladii, L. kullabergensis, L. kefiranofaciens, L. melliventris, L. pasteurii, and L. ultunensis. In these genomes, no such *fbpB* homologues were present, and the tyrosyl-tRNA synthetase gene was located directly downstream of the glucan modification gene. The G+C content of *fbpB* was consistent across species, ranging from 35.7% to 38.3%. Each of these values varied by <1% from the overall G+C content of each full genome (34.7% to 38.2%).

**FIG 3:**
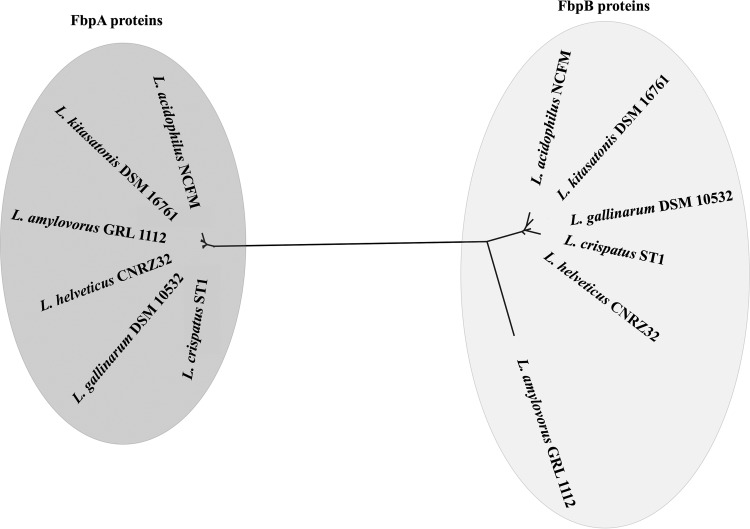
Neighbor-joining phylogenetic tree based on fibronectin-binding proteins in L. acidophilus NCFM and other *fbpB*-containing lactobacilli. FbpA and FbpB protein sequences were aligned, and the phylogenetic tree was created in Geneious 8.1.7.

An unrooted phylogenetic tree was constructed from an alignment of the two known fibronectin-binding proteins in the L. acidophilus homology group, FbpA and FbpB (Fig. 3). The two distinct clusters in the tree illustrate the low sequence identity between the two proteins. For each set of proteins, homologues share identities of at least 75%, with the exception of FbpB in L. amylovorus. The two groups contain different functional domains for binding fibronectin. In FbpA, a fibronectin-binding domain similar to that of Fbp54 in Staphylococcus aureus is considered the functional domain involved in binding. This domain is unrelated to the FN3 domain found in FbpB. The signal peptide cleavage sequence of FbpB in L. acidophilus (VQA/GT) was conserved in L. crispatus, *L*, kitasatonis, L. gallinarum, and L. helveticus. Although the signal peptide cleavage sequence varies in strains of L. amylovorus (VNA/AS), the position of the cleavage site was conserved.

### Transcriptional analysis of the *fbpB* operon.

Transcriptional data from RNA sequencing indicates that *fbpB* and the downstream tyrosyl-tRNA synthetase gene, tyrS (LBA0192), are transcribed polycistronically. Reads mapped to the L. acidophilus NCFM genome showed that transcription levels are maintained throughout the intergenic region between *fbpB* and tyrS (Fig. 2). RNA sequencing data for L. crispatus ST1, L. amylovorus GRL1112, and L. helveticus CNRZ32 also indicate polycistronic transcription. These data suggest that the downstream tyrS may operate under the control of *fbpB* regulatory sequences in all four strains.

A putative promoter sequence was identified upstream of *fbpB*. The promoter sequence consisted of a −35 element (5′-TTGTNT-3′) and a −10 element (5′-TAAAAT-3′). A putative ribosomal binding site (5′-AGGTGA-3′) was located 11 nucleotides upstream of the *fbpB* start codon. Furthermore, the promoter elements and ribosomal binding site were located upstream of *fbpB* homologues in other lactobacilli (Table 2). A Rho-independent transcription terminator sequence was identified immediately downstream of the tyrS stop codon. The predicted terminator sequence featured GC-rich palindromic stems, with a 6-nucleotide (nt) loop located upstream of a 3′ poly(T) region.

**TABLE 2:** Conservation of regulatory elements in strains with *fbpB* homologues

Strain name	−35 element	−10 element^*a*^	RBS^*a*^	Predicted SPase cleavage site^*b*^
*L. acidophilus* NCFM	TTGTTT	TAAAAT	AGGTGA	VQA/GT (24–25)
L. amylovorus GRL 1112	TTGTTT	TAAAAT	AGGTGA	VNA/AS (24–25)
L. crispatus ST1	TTGTAT	TAAAAT	AGGTGA	VQA/GT (24–25)
L. gallinarum DSM 10532	TTGTGT	TAAAAT	AGGTGA	VQA/GT (24–25)
L. helveticus CNRZ32	TTGTGT	TAAAAT	AGGTGA	VQA/GT (24–25)
L. kitasatonis DSM 16761	TTAAGT	TAAAAT	AGGTGA	VQA/GT (24–25)

a RBS and −10 element are 100% conserved.

b Cleavage site is conserved in all strains, except L. amylovorus GRL 1112. SPase, signal peptidase.

### Growth and survival of Δ*fbpB* mutant.

Stress challenge assays were performed on wild-type and Δ*fbpB* strains to investigate possible stress-sensitive phenotypes in the Δ*fbpB* mutant. Both strains were inoculated and grown for 48 h in MRS and in MRS with 0.3% oxgall, 2.5% NaCl, 10% ethanol, or 0.02% SDS. There were no significant differences between the growth rates and survival of the wild-type and mutant strains under these conditions. Additionally, both strains were mixed with simulated gastric juice as an *in vitro* assessment of gastrointestinal survival. There were no significant differences in the ability of the parent or Δ*fbpB* mutant to survive in simulated gastric juice.

### Adhesion ability of Δ*fbpB* mutant.

**FIG 4:**
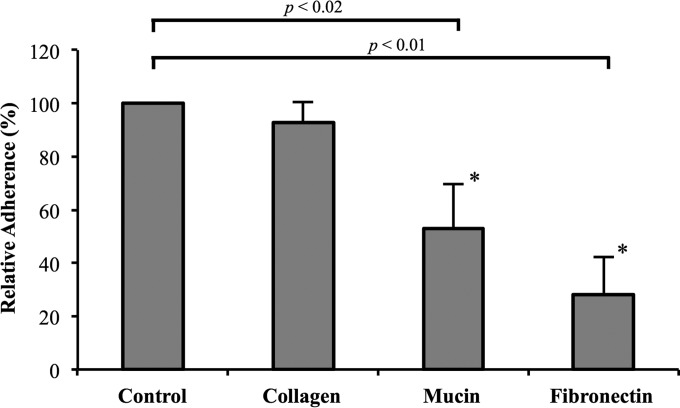
Percent adherence of Δ*fbpB* mutant (NCK2393) to fibronectin, mucin, and collagen substrates relative to the parent strain (NCK1909, 100%). The data presented here are means and standard errors from the results from three independent replicates. Asterisks indicate significance (mucin, *P* < 0.02; fibronectin, *P* < 0.01).

The Δ*fbpB* mutant exhibited a statistically significant reduction in adherence to fibronectin and mucin *in vitro* relative to that of the parent strain (Fig. 4). Adherence of the Δ*fbpB* mutant to human plasma fibronectin was reduced by 72%, and adherence to type III mucin from porcine stomach was reduced by 47%. The Δ*fbpB* mutant exhibited no significant reduction in adhesion (7.2%) to type IV collagen.

Cell surface properties of both strains were investigated using a microbial adhesion to solvents (MATS) assay. There were no significant differences between strains in affinity for chloroform, hexadecane, ethyl acetate, or decane. Both strains displayed high affinity (80 to 90%) for the nonpolar solvents hexadecane and decane. The strains also exhibited high affinity (>98%) for the acidic solvent chloroform and low affinity (50%) for the basic solvent ethyl acetate.

### S-layer thickness in Δ*fbpB* mutant.

**FIG 5:**
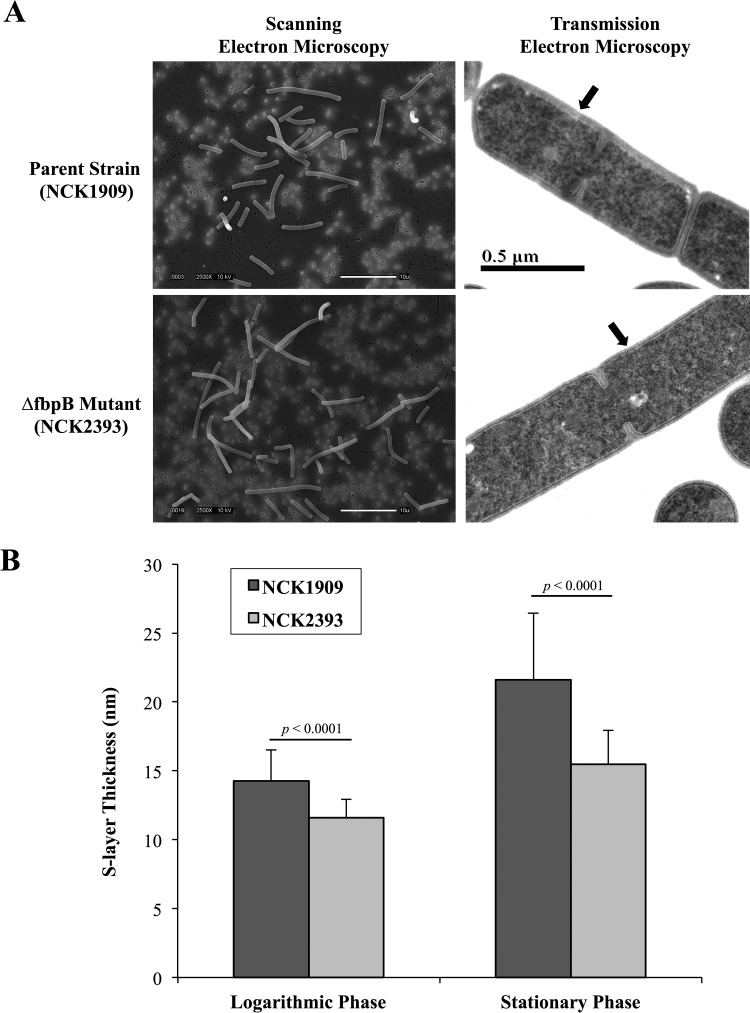
(A) Scanning electron micrographs (SEM) and transmission electron micrographs (TEM) of parent and Δ*fbpB* strains. SEM images were taken of cells at logarithmic phase (2,500×), and TEM images were taken of cells at stationary phase (10,000×). Arrows indicate S-layer. (B) S-layer thickness (in nanometers) of parent and mutant strains at logarithmic and stationary phases.

Examination of cellular morphology by transmission electron microscopy (TEM) reveals a significant reduction in S-layer thickness in the Δ*fbpB* mutant (Fig. 5). At logarithmic phase, the S-layer was thicker in the parent strain (mean, 14.25 nm; standard deviation [SD], 2.25 nm) than in the mutant strain (mean, 11.58 nm; SD, 1.34 nm). At stationary phase, the S-layer was thicker in the parent strain (mean, 21.59 nm; SD, 4.87 nm) than in the mutant strain (mean, 15.48 nm; SD, 2.47 nm). These differences were significant (*P* < 0.0001). No morphological differences between strains were visible in scanning electron micrographs (Fig. 5A).

### Extraction of noncovalently bound extracellular proteins.

**FIG 6:**
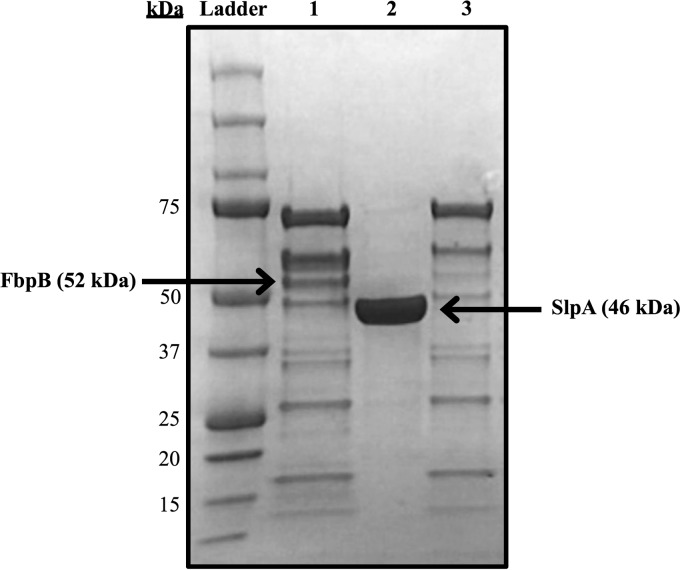
Putative SLAPs of parent and Δ*fbpB* strains (NCK1909 and NCK2393). Proteins were extracted by washing in LiCl and dialyzing in molecular porous membranes. Relative molecular masses are labeled (ladder). Lane 1, SLAPs from parent, NCK1909; lane 2, pure SlpA from parent, NCK1909; lane 3, SLAPs from Δ*fbpB* mutant. The faint band remaining at ∼52 kDa (corresponding to FbpB) in lane 3 is likely from SLAPs of similar size (SlpX, 54 kDa; LBA0864, 55 kDa).

Electrophoresis of SLAP extractions revealed nearly identical protein banding patterns in the parent strain and the Δ*fbpB* mutant (Fig. 6). The faint band remaining at ∼52 kDa (corresponding to FbpB) in lane 3 of Fig. 6 is likely from SLAPs of similar size (SlpX, 54 kDa; LBA0864, 55 kDa).

## DISCUSSION

**FIG 7:**
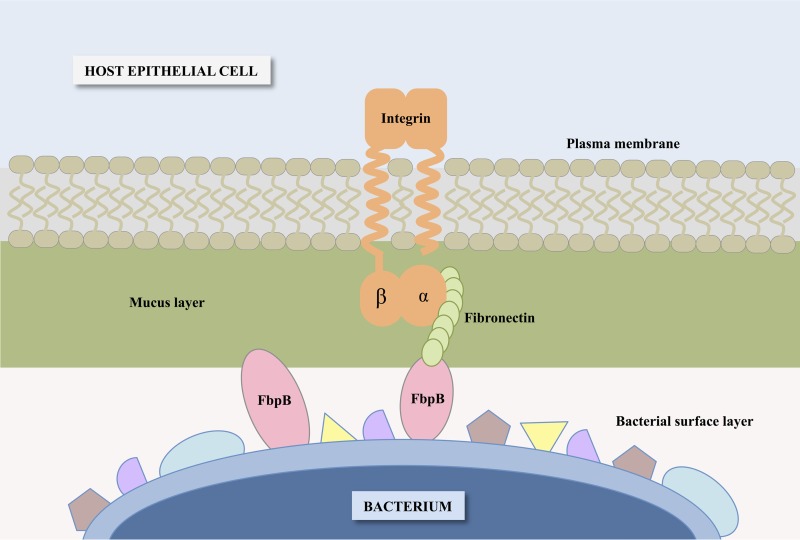
Schematic of the proposed interaction between FbpB and the bacterial cell surface, extracellular fibronectin, and mucin of epithelial cells.

In order to determine whether FbpB is involved in adhesion to fibronectin, *fbpB* was deleted from the L. acidophilus NCFM genome, and the mutant was investigated for binding to fibronectin, collagen, and mucin. The *fbpB*-deficient strain showed significant reductions in adhesion to type III mucin and human plasma fibronectin relative to the parent strain *in vitro* (Fig. 4). No reduction in adhesion to type IV collagen was observed. These results demonstrated that FbpB functions as an adhesion factor by interacting specifically with fibronectin and mucin, both of which are major components of the human intestinal lining (Fig. 7) (2, 3). Attachment to the extracellular matrix provides bacteria with an improved opportunity to interact intimately with the host gastrointestinal tract (52). As transient microbes interacting with the intestinal mucosa, probiotic strains are able to affect the host by producing inhibitory substances, stimulating the immune system, and competing with pathogens for adhesion sites and nutrients (20). Transmission electron micrographs reveal a significant decrease in S-layer thickness in the Δ*fbpB* mutant compared to that of the parent strain (Fig. 5). S-layer thickness measurements ranged from 11.58 nm to 21.59 nm, which is consistent with previous measurements describing S-layers as being 5- to 25-nm thick (53). Previous studies have demonstrated that knockouts of the major S-layer component (SlpA) significantly reduce the adhesion of lactobacilli to intestinal cells and components of the extracellular matrix *in vitro* (30, 54, 55). These results suggest that the loss of FbpB may indirectly reduce adhesion ability by affecting the size of the S-layer. Aberrant S-layer thickness may further reduce adhesion ability by disrupting the attachment of SLAPs that are typically embedded in the S-layer. Overall, the cell morphology of NCK2393 was not affected by the deletion of *fbpB*, as shown in scanning electron micrographs (Fig. 5A). Electrophoresis of noncovalently bound extracellular proteins provides a visualization of the SLAP profiles for each strain (Fig. 6). Excluding the loss of FbpB, the banding patterns suggest no differences in the composition of the exoproteome between the mutant and parent strains.

Assays for growth rate and survival in various stress challenge media, including ethanol, sodium chloride, bile, sodium dodecyl sulfate, and simulated gastric juice, showed no difference between the mutant and parent strains. As FbpB is not predicted to be involved in cell growth, division, or nutrient acquisition, no variation in these phenotypes was anticipated in the mutant.

The results of the assay for microbial adhesion to solvents demonstrate that the cell surface of the mutant exhibited no significant change in hydrophobic properties relative to the parent strain. The high affinity of both strains for nonpolar solvents indicates that L. acidophilus has a hydrophobic cell surface, confirming data from a previous study by Goh et al. (38). The affinities of the parent and Δ*fbpB* strains for chloroform and ethyl acetate are also consistent with this study. A nonsignificant increase in affinity for ethyl acetate was observed in the Δ*fbpB* mutant, indicating that the cell surface of the FbpB-deficient mutant may have stronger acidic characteristics relative to those of parent strain. The absence of FbpB (pI, 10.01) on the cell surface of the Δ*fbpB* mutant may contribute to a slight increase in acidic cell surface properties, which might in turn affect cell attachment.

Sequence similarity searches indicate that genes with homology to *fbpB* are present in the genomes of only five species, all within the L. acidophilus homology group (Fig. 1). No homologous genes were detected outside this phylogenetic subset of lactobacilli. Notwithstanding the high level of sequence identity, these five FbpB homologues also share conserved regulatory sequences, genomic context, and expression levels. Furthermore, an alignment of known fibronectin-binding proteins in lactobacilli shows a distinct clustering of FbpB sequences. Taken together, these data suggest that FbpB proteins comprise a unique class of fibronectin-binding proteins found in only a subset of Lactobacillus species. Unlike the other homologues, the *fbpB* sequence in L. amylovorus is truncated by 90 amino acids. The signal peptidase cleavage site and collagen-binding domain are present, but the entire FN3 domain region of the sequence is absent. In this study, the sequence is considered a homologue because of overall sequence similarity and domain conservation. However, the absence of the fibronectin-binding domain suggests that this protein may play a more limited role in the bacterial attachment of L. amylovorus to fibronectin.

A conserved genomic context was detected in the genomes of all six species with *fbpB* sequences (Fig. 2). Analysis of whole-genome sequences reveals that *fbpB* is flanked by the same three upstream genes and two downstream genes in the genomes of L. acidophilus, L. amylovorus, L. crispatus, L. gallinarum, L. helveticus, and L. kitasatonis. We also detected that the genomes of 13 additional lactobacilli feature the aforementioned conserved contextual genes, despite the absence of a medial *fbpB* gene. This suggests that during the evolution of some Lactobacillus species, *fbpB* was either acquired or lost, while the arrangement of neighboring genes was fully conserved.

L. delbrueckii, the type species of the L. acidophilus homology group, does not contain an FbpB homologue (51). L. delbrueckii is also deficient in SlpA, the major constituent of the S-layer. In fact, FbpB homologues are found exclusively in species predicted to form S-layers (46). The clear association of FbpB with S-layer-forming species is consistent with the recent classification of FbpB as a SLAP in L. acidophilus (37). The absence of genes with homology to *slpA* or *fbpB* in L. delbrueckii suggests that horizontal gene transfer may have led to the acquisition of these genes after divergence from L. delbrueckii. Interestingly, all but one of the species with *fbpB* homologues (L. helveticus) were originally isolated from the intestines of mammals. In these environments, the ability to adhere to extracellular matrices provides an evolutionary advantage. After the initial acquisition of *fbpB*, closely related species may have acquired the gene through vertical transfer. Alternatively, the gene may have evolved convergently as a result of adaptation to similar ecological niches.

Conserved levels of intergenic transcription between *fbpB* and the downstream tyrosyl-tRNA synthetase gene suggest the two genes are transcribed polycistronically (Fig. 2). The tyrS gene encodes an essential protein responsible for loading tRNA molecules with tyrosine. The resulting tyrosyl-tRNA molecule can transfer tyrosine from tRNA onto a growing peptide. Thus, the tyrosyl-tRNA synthetase gene is necessary for translation. Because the in-frame deletion of FbpB in this study left behind the entire upstream and downstream intergenic regions, the expression of TyrS is not predicted be affected by *fbpB* deficiency. Although they appear to be cotranscribed, FbpB and TyrS have no apparent overlap in function. Interestingly, FbpB has a tyrosine composition of 7.3%, nearly twice the average genome-wide tyrosine composition. Further research on the regulation and function of FbpB and TyrS may elucidate the purpose of their transcriptional relationship.

In this study, we show that NCK2393 (Δ*fbpB*) cells exhibit a significant reduction in adherence to mucin and fibronectin *in vitro*. Thus, FbpB may potentially function as an adhesion factor by interacting with fibronectin components of the ECM and the mucus layer of host intestinal epithelial cells. To confirm the findings, the adhesion ability of the Δ*fbpB* strain relative to the parent strain should be assessed *in vivo*. Previous work has demonstrated that an insertional knockout of *slpA* from L. acidophilus NCFM significantly reduces adhesion ability to intestinal epithelial cells *in vitro* (30). The recent identification of SLAPs has enabled researchers to consider whether SlpA is solely responsible for the loss of adherence ability observed in the Δ*slpA* strain (37). Current efforts are establishing a new model whereby SlpA and coassociated SLAPs work cooperatively to mediate adhesion. By anchoring extracellular proteins with substrate-specific adhesive properties, the S-layer and its SLAPs appear to play an important role in adhesion and subsequent host signaling to the immune system of the gastrointestinal mucosa.
